# Comment on Colombo et al. Managing Retinitis Pigmentosa: A Literature Review of Current Non-Surgical Approaches. *J. Clin. Med.* 2025, *14*, 330

**DOI:** 10.3390/jcm14082553

**Published:** 2025-04-08

**Authors:** Filippo Confalonieri, Vanessa Ferraro, Alessandra Di Maria

**Affiliations:** Department of Ophthalmology, IRCCS Humanitas Research Hospital, Rozzano, 20089 Milan, Italy; vanessa.ferraro@humanitas.it (V.F.); alessandra.di_maria@humanitas.it (A.D.M.)

It is with great interest that we read the article by Colombo et al. “Managing Retinitis Pigmentosa: A Literature Review of Current Non-Surgical Approaches” [1] recently published in the *Journal of Clinical Medicine*. The authors have compiled an insightful review that underscores the significance of non-surgical interventions, such as visual training, photoprotection, and psychological counseling, in improving the quality of life for patients with retinitis pigmentosa (RP). The emphasis on a multidisciplinary care approach is commendable and highlights the importance of comprehensive patient management while curative therapies remain limited.

We would like to draw attention to the considerable overlap between this article and our recently published systematic review, “Retinitis Pigmentosa and Therapeutic Approaches: A Systematic Review”, which appeared in the *Journal of Clinical Medicine* in 2024 (Confalonieri et al. *J. Clin. Med.* **2024**, *13*, 4680. doi:10.3390/jcm13164680) [2]. Our work presented a systematic synthesis of emerging therapeutic modalities, encompassing gene therapy, mesenchymal-cell-based approaches, and supplementary interventions, including nutritional supplementation and neuroprotective agents. Importantly, our review also highlighted the gaps and heterogeneity in the current evidence base, providing detailed recommendations for future research to refine treatment strategies and enhance patient outcomes.

While we recognize that the two articles focus on slightly different aspects of RP management, both share a common goal of addressing the needs of patients through a multidisciplinary approach and exploring the potential of non-surgical and supplementary interventions. Specifically, our review emphasized the emerging roles of gene- and cell-based therapies, as well as the integration of these advanced treatments with established non-surgical approaches. Colombo et al.’s article, on the other hand, places greater emphasis on established practices such as visual aids, photoprotection, and psychological counseling. This difference highlights complementary perspectives: while Colombo et al. focus on optimizing existing non-surgical interventions, our work expands on novel therapeutic frontiers that are still under investigation.

Given the temporal proximity and thematic alignment of the two works, we believe that the inclusion of our review in Colombo et al.’s article would have provided readers with a more comprehensive perspective on the topic, particularly with regard to the heterogeneity of evidence and the emerging role of gene- and cell-based therapies in RP management. Additionally, our review underscores the importance of addressing the interplay between established and experimental interventions to develop a cohesive care framework.

We also concur with Colombo et al.’s observation that non-surgical interventions, such as visual aids, photoprotection, and psychological counseling, play a crucial role in preserving patients’ functional abilities and alleviating the emotional burden of progressive vision loss. However, as we emphasized in our systematic review, these approaches require further validation through well-designed, large-scale studies to establish their efficacy and long-term safety. Moreover, we advocate for the integration of these interventions with emerging therapies, such as gene therapy and mesenchymal-cell-based treatments, to develop a more holistic care framework for RP patients.

In conclusion, we commend Colombo et al. for their valuable contribution to the growing body of research on RP management. By addressing the differences and complementarity between our respective works, we hope this correspondence highlights the importance of acknowledging and building upon recent research to advance the understanding and treatment of this complex condition. We believe that fostering collaboration and dialogue among researchers will accelerate progress and ultimately benefit patients.
